# Imiquimod for Anal High Grade Intraepithelial Neoplasia: A Systematic Review

**DOI:** 10.1007/s11912-025-01675-1

**Published:** 2025-05-19

**Authors:** Niccolò Gallio, Mario Preti, Elena Casetta, Andreia Albuquerque, Pedro Vieira-Baptista, Fulvio Borella, Federica Bevilacqua, Camilla Cavallero, Massimiliano Mistrangelo, Alberto Revelli

**Affiliations:** 1 Obstetrics and Gynecology Unit 2, Department of Surgical Sciences, “City of Health and Science University Hospital”, S. Anna Hospital, University of Turin, Via Ventimiglia, 3 10126 Turin, Italy; 2 Obstetrics and Gynecology Unit 1, Department of Surgical Sciences, Sant’ Anna Hospital, University of Torino, Turin, Italy; 3 Gastroenterology Department, Fernando Pessoa Teaching Hospital, Porto, Portugal; 4 Precancerous Lesions and Early Cancer Management Research Group RISE@CI-IPO (Health Research Network), Portuguese Oncology Institute of Porto (IPO-Porto), Porto, Portugal; 5 Department of Gynecology-Obstetrics and Pediatrics, Faculdade de Medicina da Universidade Do Porto, Porto, Portugal; 6 Lower Genital Tract Unit, Centro Hospitalar de São João, Porto, Portugal; 7 Surgical Sciences Department, University of Torino, Città Della Salute E Della Scienza Di Torino, Turin, Italy

**Keywords:** Imiquimod, Anal Intraepithelial Neoplasia, HPV lesion, Anal HSIL, Topical therapy, Intra-anal HSIL, Perianal HSIL

## Abstract

**Introduction:**

This study aimed to investigate the efficacy of imiquimod in Anal High Grade Squamous Intraepithelial Lesion (HSIL).

**Methods:**

Electronic databases (Pubmed, MEDLINE, EMBASE and Cochrane Library databases) were searched from inception until December 2024 for articles reporting imiquimod as a treatment for anal HSIL.

**Results:**

Five studies were identified (2 randomized controlled trials and 3 prospective non-randomized studies), containing data on 126 men of have sex with men living with HIV with anal HSIL. Most studies contained significant bias which prevented direct comparison. Reported complete response (CR) rates ranged between 14.3–78.6%, and 21.4–67% partial response (PR) rates of 3-weekly application for 16 weeks imiquimod course. A second course of imiquimod led to incremental response (CR 15–23.8%, PR 19–30%). Perianal HSIL showed superior response rates compared to intra-anal lesions (perianal HSIL CR ranging from 71.4 to 100%, intra-anal HSIL CR from 10.8 to 33.3%).

**Discussion:**

In our systematic review we summarized the literature regarding imiquimod use for anal HSIL treatment, both perianal/intra-anal. Imiquimod can be proposed as a safe treatment of anal HSIL, and perianal HSIL may benefit more from imiquimod treatment. However, anal HSIL recurrence rates were high, and there are no long-term data on its efficacy. No studies investigated the role of imiquimod in women or in HIV- patients.

**Conclusion:**

Imiquimod can be proposed as a safe option for treatment of anal HSIL.

## Introduction

Anal high-grade squamous intraepithelial lesion is the precursor of anal squamous cell carcinoma (ASCC). Anal cancer progresses similarly to cervical cancer, with reported incidence rates markedly increasing to 2–3 cases per 100.000 people/year worldwide [1]. HPV infection is the major etiological factor in ASCC development, being detected in more than 90% of specimens, and with HPV 16, 18 and 33 the most frequent genotypes [2].

Risk factors for AIN development include human immunodeficiency virus (HIV) infection, receptive anal intercourse [3], cigarette smoking, and anal warts [4]. Some specific populations are at higher risk of AIN, notably people living with HIV (PLWH), men who have sex with men (MSM), women with previous diagnosis of vulvar cancer or precancer, and solid organ transplant recipients [5, 6].

Analogously to cervical oncogenesis, AIN is graded into low-grade (LSIL or AIN 1) and high-grade squamous intraepithelial lesions (HSIL, encompassing p16 positive AIN 2 and AIN 3) [7].

The primary focus of treatment is addressing potentially precancerous anal HSILs to reduce the risk of progression, which reaches 14.1% in PLWH compared to 3.2% in people without HIV [8]. Anal HSIL treatment is recommended based on the results of the ANCHOR study, which demonstrated that treatment of HSIL reduces the incidence of ASCC in PLWH [9] compared to active monitoring. Several treatment modalities have been described [10] with the choice largely driven by local availability and clinician preference, due to the lack of randomized controlled trials (RCTs). Treatment options include excisional surgery, ablative treatments (e.g., infrared coagulation, cryotherapy, LASER ablation) or topical treatments (e.g., imiquimod, fluorouracil, cidofovir, and trichloroacetic acid).

Imiquimod is an immune response modifier licensed for use in the topical treatments for certain skin conditions, such as genital warts, actinic keratosis, and superficial basal cell carcinoma [11]. The mechanism of action of imiquimod involves activating the innate immune response by binding to toll-like receptor 7 (TLR7) on immune cells, particularly dendritic cells and macrophages. This binding triggering the production of various cytokines, including interferon-alpha (INFα), tumor necrosis factor-alpha (TNFα), and interleukins, which play a crucial role in orchestrating an antiviral and antitumor immune response^9^, with migration of Langerhans cells to local lymph node to activate immune cells, in particular Th- 1 T-helper response, natural killer cells and macrophages. These cytokines enhance the activity of immune cells that target abnormal or HPV infected cells. Additionally, imiquimod is successfully used as an off label treatment in a number of conditions, including cervical, vulvar, vaginal SILs, and extramammary Paget disease [11–15]. Given imiquimod effectiveness as an off-label treatment of many HPV-related lesions, it has also been used in anal HSIL, with emerging data suggesting it to be a convenient, self-administered topical treatment.

The aim of the present systematic review is to investigate the role of imiquimod in anal HSIL treatment and its potential side effects.

## Methods

This systematic review was conducted in accordance with the Preferred Reporting Items for Systematic Reviews and Meta-Analyses (PRISMA) Statement.

### Search Methods

A literature search was performed in the PubMed, MEDLINE, EMBASE and Cochrane Library databases for published papers from inception until December 2024 Searching was performed using the following keywords: “imiquimod”, “anal intraepithelial neoplasia” OR “anal squamous intraepithelial lesion” OR “anal dysplasia”, AND (“treatment” OR “imiquimod”OR “topical therapy”).

All articles’ titles and abstracts reporting imiquimod treatment for anal HSIL intraepithelial neoplasia were individually screened and reviewed by two authors (FB, NG). If there was disagreement on inclusion, a consensus was obtained after discussion.

Letters to the editor and papers with incomplete or uninterpretable data were excluded during the screening process. Articles where imiquimod was used in combination with other treatments were also excluded. Systematic reviews, literature reviews, society statements were also excluded, since they provided synthesis of evidence of already selected studies. Papers in languages other than English were excluded.

Full-length articles were obtained as the second step of screening. Reference lists from the above articles were subsequently screened for other potentially relevant articles. Two authors (FB, NG) did the full-text screening, and clinical information, dose, frequency of application, dosage and formulations of imiquimod, and treatment outcomes were extracted from the selected articles, and listed in an Excel 2024, Microsoft Corporation, Redmond, Washington, USA spreadsheet table.

Outcomes measures in anal HSIL imiquimod use were: complete/partial response, recurrence, persistence, and side effects. Since these measures may be used differently across studies, they were specified in text and tables if available on the original paper (histological, cytological or clinical). Otherwise, the treatment response was extracted from authors’ comments. Complete response (CR) was defined as histological or cytological evidence of total regression, and in the absence of these criteria, on clinical examination. The definition of partial response (PR) varied across studies and was further specified in the text and tables.

## Results

### Study Selection

**Fig. 1:**
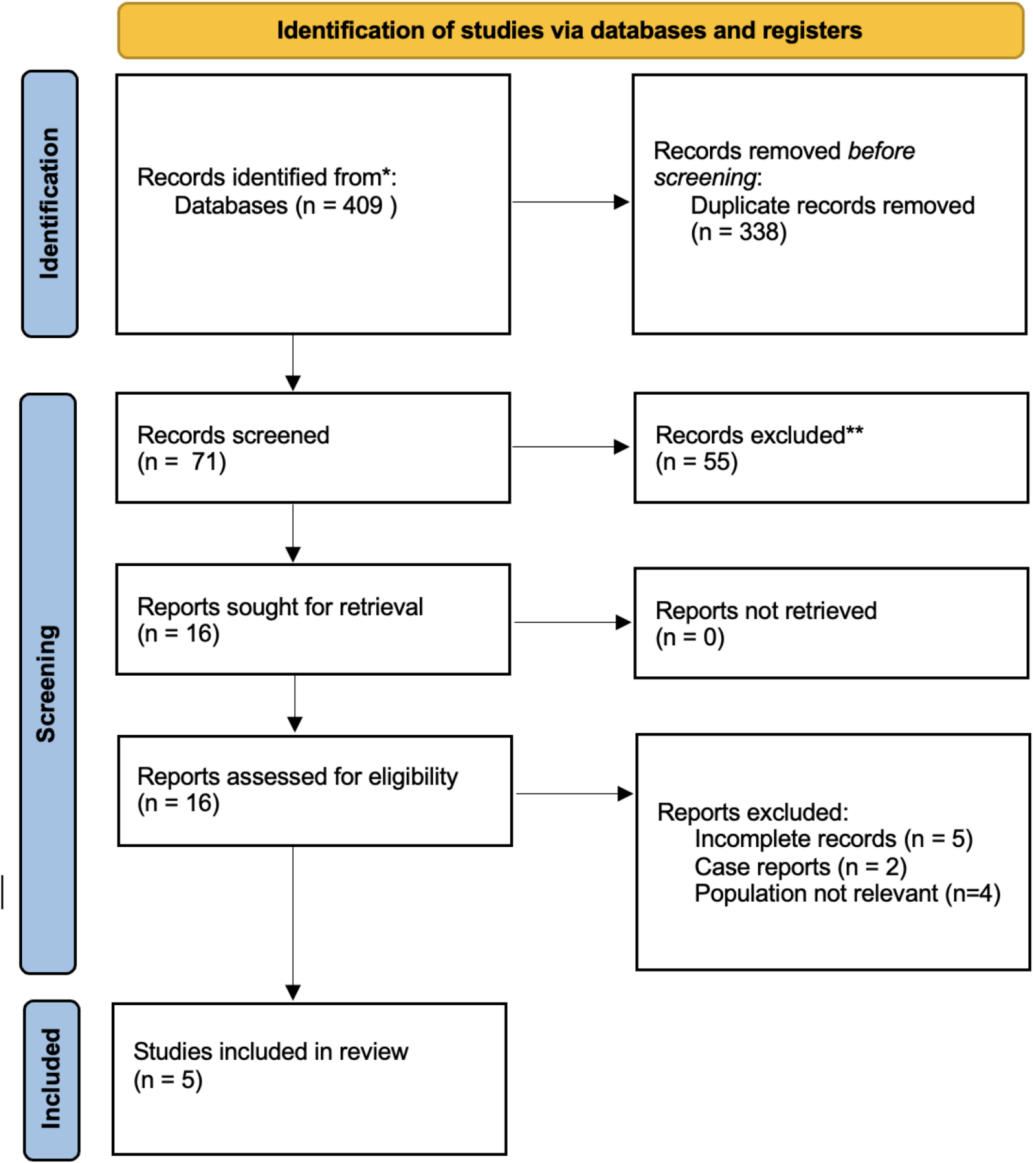
Systematic review flowchart

Figure 1 shows the selection flow chart of relevant studies. A total of 409 articles were retrieved, and 5 articles were finally selected for inclusion in the present review (study selection process is summarized in Fig. 1).

### Summary of the Selected Studies

#### Study and Population Characteristics

The systematic search included 5 studies (3 prospective non-randomized studies and 2 RCTs), with study selection flow chart in Fig. 1. The main characteristics of the studies included are summarized in Table 1. The study population only included MSM living with HIV.

**Table 1:** Summary of articles included in systematic review

	Authors				
	Wieland	Fox	Richel	Cranston	Van der Snoek
Year of publication	2006	2010	2013	2018	2015
Study design	Prospective non-randomised open-label pilot study	Double blind RCT	Open-label RCT	Prospective, non-randomised open-label pilot study	Prospective open-label pilot study
Total number analysed	22	53 (28 in Imiquimod arm and 25 in placebo arm)	54	9	44
Study population characteristics (MSM/TW/Cis/etc)	MSM	MSM	MSM	MSM	MSM
Male	100%	100%	100%	100%	100%
Mean age	43 (31-69)	42 in Imiquimod, 42 in placebo	47	46	-
HIV +	100%	100%	100%	100%	100%
HPV DNA positive	100% High Risk HPV			100%	
HAART	93%		86%	100%	90.5%
Anal HSIL	64% (14)	100%	57% (31/54)	100%	100%
Perianal HSIL	11	28	11	-	7
Intraanal HSIL	3	-	50	9	37
Intervention	Imiquimod 5% 3 times a week for 16 weeks	Imiquimod 5% 3 times a week for 16 weeks (28)	Imiquimod 5% 3 times a week for 16 weeks (54)	Imiquimod 5% 3 times a week for 9 weeks	Imiquimod 5% weekly 5 consecutive days for 16 weeks, if no responde or PR another 16 weeks course
Comparison	-	Placebo (25)	5-FU for 16 weeks (48) or monthly electrocautery for 4 months (46)	-	
Type of Imiquimod	Self-applied 5% Imiquimod cream (perianal AIN) or suppositories (intraanal AIN)	Self-applied Imiquimod	Self-applied Imiquimod	Self-applied Imiquimod	Self-applied Imiquimod
Dosage/formulation	-	6.25 mg /cream	6.25 mg /cream	12.5 mg /cream	6.25 mg /cream
**Outcomes for HSIL**					
Complete response	78.6% (11/14)	14%. 46.4% in Imiquimod group, 4% in Placebo. At 33 months, 61% in Imiquimod group.	21% (5/24)	33%	20% (9) at 16 weeks, 66% (29) at 32 weeks
Partial Response	21.4% (3/14)	29% in Imiquimod, 0% in Placebo	25% (6/24)	67%	25% at 16 weeks, 39% at 32 weeks
No response			54% (13/24)		
Recurrence at end of follow up	14.3% (2/14)	39%	-	-	10%
Persistence	7.1% (1/14)	-	48% in Imiquimod, 60% in 5-FU, 33% in Electrocautery	-	65% after 32 weeks
Time to recurrence	-	-		-	
**Follow Up**					
Duration (months)	9.87	36	4.5 (response), 16.5 (recurrence)	11 days	12
Compliance	79%	83%	91%	90%	100%
Progression to invasion	0	2% (2 in placebo arm)	0	0	0
Outcomes for perianal HSIL	81.8% CR (9/11), 18.2% PR (2/11)	-	100% CR (9/9)		71.4% CR (5/7) and 18.6% PR (2/7) after 16 weeks
Outcomes for intraanal HSIL	33.3% CR (1/3),67.7% PR (2/3)		22% CR (9/41), 15% PR (6/41), No response 63% (26/41)		10.8% CR (4/37) and 20.5% PR at 16 weeks,
Level of evidence	4	1b	1b	4	4

One randomized double-blind controlled trial [16] by Fox et al. compared imiquimod to placebo in treating intra-anal HSIL in 53 MSM living with HIV: 28 were assigned to active treatment and 25 to placebo. The imiquimod schedule consisted of 3 applications/week for 16 weeks and dosing was half a sachet intra-anally, to reduce potential systemic absorption and side effects. The CR rate was 14.3%; 29% had PR and in 57.1% the lesions persisted in the imiquimod arm, while the placebo arm showed only 4% CR and 96% of persistent lesions. After trial completion, partial responders, non-responders, and those in the placebo arm were offered an open-label treatment with imiquimod with the same schedule and dosing (21 patients in total, of whom 12 were originally from the placebo arm), resulting in a total of 23.8% CR and 19% PR rates. Thus, in total a sustained CR was observed in 61.7% (29/47 patients) over a mean duration of follow-up of 30 months. Two patients out of 53 developed ASCC (3.8%), one originally assigned to the placebo group after a few weeks into the treatment arm, which may be considered a prevalent case, in which the diagnosis was missed at study entry. The other one that progressed to ASCC was in the open label group, but no further information is available from the paper.

Wieland et al. published a prospective non randomized open-label pilot study that included 22 MSM living with HIV (18 perianal and 4 intra-anal AIN) compliant to therapy [17]. Out of the 22 patients, 64% (14) had histology-proven anal HSIL (11 perianal and 3 intra-anal). Imiquimod treatment consisted of self-applied 5% cream for perianal HSIL or suppositories for intra-anal HSIL 3 times a week for 16 weeks. After treatment, 78.6% (11/14) had a CR and 21.4% (3/14) had residual AIN- 1. After a mean 9.87 months of follow up after treatment, 10 maintained a CR, 1 recurred as HSIL, 2 recurred as LSIL, while 1 that had residual AIN at end of treatment had persistent LSIL. With respect to the 11 cases of only perianal HSIL, 81.8% (9/11) had a CR and 18.2% (2/11) had residual AIN 1 after treatment. After a mean of 11.1 months of follow up, 81.8% (9/11) maintained a CR, while 1 recurred as HSIL at 12 months and 1 as LSIL at 6 months.

An open-label RCT by Richel et al. compared imiquimod, topical 5-fluorouracil and electrocautery for the treatment of AIN in 156 MSM HIV + [18]. Anal HSIL was present in 57% of patients, while 43% had LSIL. 54 patients were randomly assigned to imiquimod, 48 to topical fluorouracil, and 46 to electrocautery. Imiquimod was administered three times weekly for 16 weeks (6.25 mg). For perianal HSIL, half a sachet was the standard dose, while in intra-anal HSIL the volume was increased by an indifferent cream and applied with a standard applicator. Regarding only anal HSIL, a CR was recorded in 21% (5/24 patients, per protocol analysis), a PR in 25% (6/24) and no response in 54% (13/24). Furthermore, response rates were higher for perianal HSIL than for intra-anal ones, and imiquimod showed superior outcomes compared to other treatments: a CR of 100% (9/9) for perianal HSIL and a 22% CR (9/41), 15% PR (6/41) and no response 63% (26/41) for intra-anal HSIL.

Van der Snoek et al. [19] included 44 anal HSIL in HIV + MSM, 15.9% with perianal HSIL in and 84.1% with intra-anal HSIL. Patients were instructed to apply imiquimod during five consecutive nights per week for 16 weeks (6.25 mg). For perianal HSIL, half a sachet was the standard dose, while in intra-anal HSIL the volume was increased by an indifferent cream and applied with a standard applicator. After 16 weeks of treatment, a CR of 20% and PR of 25% were recorded, with an overall response rate of 45%. Perianal lesions showed an overall response rate (RR) of 100% (71% CR and 29% PR), while intra-anal HSIL an overall RR of 35% (11% CR and 24% PR). An additional 16-weeks course of treatment was offered to another 20 partial responders and non-responders, which resulted in more responders (45%; 15% CR and 30% PR). Thus, after 32 weeks, an overall response rate of 66% was reported, 59% in intra-anal HSIL and 100% in perianal HSIL. After 12 months of follow-up, 3 patients (8%) experienced HSIL recurrence.

A prospective non-randomized open-label pilot trial enrolled 9 HIV + MSM with anal HSIL treated with imiquimod 3 times a week for 9 weeks. A CR of 33% was achieved and 7 out of 20 lesions in 9 participants (35%) downgraded from HSIL to LSIL [20]. No effects were observed in cytokine gene expression, HIV 1 DNA/RNA levels, or HPV load or types.

#### Type of Regimen

The frequency of application was 3 times weekly in almost all studies, following a schedule similar to that for condyloma [16, 17, 21, 22]. One study increased frequency of application to 5 times weekly, but apparently this did not lead to superior outcomes in terms of responses [19]. The treatment duration was 16 weeks [16, 17, 19, 21] in all but one study, in which it was of 9 weeks [22].

An additional 16-weeks treatment was offered to partial responders or non -responders in two studies, with an increase in the response rate [16, 19].

#### Type of Treatment

Imiquimod was mainly self-applied in cream form for both intra-anal and perianal disease in most studies [16, 19, 21, 22].

In two studies, Wieland et al. and Van der Snoek et al. suppositories for intra-anal disease were used [17, 19].

The dosage of imiquimod was typically 6.25 mg per application (half-sachet) [16, 17, 19, 21] while one study [22] allowed the use of a whole sachet, but with a shorter treatment duration.

#### Side Effects

Fox et al. reported that only one patient discontinued the treatment due to side effects (out of 28 in the imiquimod arm), but the specific type of side effect was not detailed [16].

In the RCT by Richel et al. side effects were reported by 91% of patients treated with imiquimod, with 9% of patients discontinuing treatment due to its severity [21]. Grade 1–2 side effects were reported by 47% of the patients and grade 3–4 by 43% (mainly pain, bleeding, itching, flu-like symptoms, and fatigue).

Cranston et al. documented 7 adverse events in 5 out of 9 participants: 4 cases of anal irritation, 1 of chills, 1 of fatigue, and 1 hemorrhoid flare [22].

Reported adverse events were erythema in all patients and further events in 11/22 compliant patients in Wieland’s paper [17]. Of these, 7 patients (32%) experienced mild erosions and 1 severe erosion (due to overdosage). Four patients (18%) had influenza-like symptoms within the 2 first weeks of therapy.

In the Van der Snoek et al. study adverse effects were present in 95.5% [19], with the most frequently reported being pain during defecation, burning sensations, mood swings, and fatigue, while flu-like symptoms were rarely observed (< 5%). Side effects did not differ between 3-days and 5-days schedules [19]

## Discussion

Since the publication of the ANCHOR study, it has been established that treating anal HSIL in people living with HIV individuals who are 35 years or older reduces the risk of progression to invasive ASCC. In this trial, 4446 participants with anal HSIL were randomly assigned to either treatment (office-based ablative procedures, ablation or excision under anesthesia, or the administration of topical 5-fluorouracil or imiquimod) or active monitoring group. The rate of progression was 57% lower in the treatment group (173 per 100,000 person-years *vs*. 402 per 100,000 person-years). However, the study was not designed to compare efficacy between different treatments, and most guidelines do not specify the best treatment for anal HSIL.

In our systematic review we summarized the literature regarding imiquimod use for anal HSIL treatment, both perianal/intra-anal. Imiquimod can be proposed as a safe treatment of anal HSIL, with reported complete response rates ranging between 14.3–78.6%, and 21.4–67% partial response. Perianal HSIL may benefit more from imiquimod treatment, since they show superior response rates compared to intra-anal lesions (perianal HSIL CR ranging from 71.4% to 100%, intra-anal HSIL CR 10.8%− 33.3%).

Application of imiquimod in AIN is still limited since it is an off-label treatment, it is not approved by the FDA or other regulatory agencies, and the clinical evidence is limited. Imiquimod has been found also to be an alternative therapy to surgery for Cervical Intraepithelial Neoplasia (CIN) and Vaginal Intraepithelial Neoplasia (VaIN), even if evidence is still limited.

Perianal HSIL seems to respond better to topical imiquimod treatment [21, 23] compared to intra-anal HSIL. Data regarding perineal HSIL should interpreted cautiously because low number of included patients and heterogeneity between studies. However, this may be explained by several factors, including the easier application of cream on perianal skin rather than intra-anal mucosal spreading, which may results in suboptimal exposure of the anal folds. Additionally, the different types of epithelia may affect the absorption rate, even if the thinner barrier of mucosal tissues theoretically would allow for more effective drug delivery compared to the keratinized epithelium. Skin penetration studies have shown that stratum corneum represents a modest barrier itself and most penetration properties are related to skin solvent uptake [24]. Another limitation may be effective cream spread into anal squamocolumnar junction (SCJ), or pectinate (dentate) line, where most lesion are located [25], similarly to cervix uteri. Anal SCJ may reside up to many centimeters up the anal canal, and difficult to reach. In this regard, a second course of Imiquimod seems to increase response rates [16, 19], without increasing adverse effects. Thus, patients should be advised that a second four months course may be required to clear lesions (CR 15–23.8%, PR 19–30% [16, 19]), and that a strict clinical follow up is mandatory. Furthermore, increasing the frequency of application to 5 weekly does not seem to be more effective compared to the classical three times a week [26]. These evidences underline that intra-anal and perianal lesions are different entities even if they affect close structures, and involve different tissue types (keratinized vs. non-keratinized) and microenvironments, which can influence their behavior and progression. Thus, further studies should focus on the efficacy of intra-anal administration, and on new ways to improve responses.

Building on these findings, the role of Imiquimod in treating other HPV-related lesions has been explored in various anatomical sites. For instance, its efficacy has been demonstrated in vulvar HSIL (VHSIL), where it has shown comparable outcomes to surgical interventions. A randomized non-inferiority trial involving 110 patients with vulvar HSIL (VHSIL) found that 80% of those treated with imiquimod achieved a complete clinical response, compared to 79% in the surgery group, demonstrating that imiquimod is non-inferior to surgical intervention [27]. Additionally, there were no significant differences in HPV clearance, adverse events, or treatment satisfaction between the two groups. These findings suggest that imiquimod is a safe, effective, and well-accepted alternative to surgery for women with VHSIL, and it can be considered as a first-line treatment.

Regarding the efficacy of imiquimod in reducing the burden of HPV infection, data from studies are discordant. Kreuter et al. reported in their pilot study [28] that a 16-weeks treatment course can reduce significantly lesional high-risk HPV load and p16 expression, and also Wieland et al. [23] reported a drop in viral loads and number of HPV genotypes after therapy, especially in complete and partial responders. Long-term follow-up [29] showed persisting lower levels of HPV loads and still significantly lower than before therapy, and a lower number of HPV genotypes. However, a more recent report from Cranston et al. [22] showed no change in cytokines levels or in number of HPV genotypes detected before and after treatment, but it must be taken into account that a shorter 9-week course was used. Pelletier et al. also reported no changes in HPV DNA positive rates after 6-weeks treatment [30]. These data should be interpreted cautiously, given the low number of patients and different treatment lengths, which may impair comparison and conclusions.

Regarding adverse events, local adverse effects such as irritation and erythema are common while systemic adverse events such as flu-like syndrome, fatigue and chills are less frequently reported [31]. In most cases, a temporary interruption of treatment was sufficient to resolve these issues, and discontinuation of treatment was required in only a few patients. No serious adverse effects were reported. Indeed, local side effects are associated to CR in vulvar HSIL and basal cell carcinoma treatment [32, 33]. Anti-inflammatory drugs do not interfere with imiquimod action [34] and can be administered in case of local pain or fever, effectively controlling symptoms.

Also, progression to invasion was a limited finding in the included studies, and may represent a missed diagnosis at trial entry. Thus, it is crucial to perform adequate multiple mapping biopsies before imiquimod administration. Furthermore, since only a limited fraction of anal HSIL may progress to ASCC, new biomarkers are highly needed in order to stratify cancer risk. Gene methylation has been proposed with good results, in terms of risk stratification, with increasing methylation levels at increasing progression risks, and with good diagnostic accuracy in anal HSIL detection [35].

Regarding medium term response after imiquimod treatment, a follow-up study of a previous pilot trial [17] was published by Kreuter [29]. It included 19 HIV + MSM who had achieved CR in a previous study [17] with a mean follow up time of 30.3 months. Of these, 74% (14/19) had normal cytology and normal clinical status evaluated by high resolution anoscopy at previously treated sites. However, 26% experienced a recurrence after 24.6 months and 58% developed new cytological abnormalities in previously normal untreated anal region. Additionally, a prolonged decrease in the number of HR-HPV types and viral loads was observed in patients with CR after treatment. However, the study did not stratify between anal LSIL and HSIL, thus limiting the usefulness of these data for purpose of the present review.

Our systematic review has some limitations: the overall quality of evidence was weak, with most data coming from uncontrolled or retrospective studies, showing a significant risk of bias. Data regarding only anal HSIL were extracted, but it was not always specified nor stratified according the intra or perianal localization.

In particular, no included studies focused on the role of imiquimod in HIV-negative patients, nor in women. We may assume that the results may be more favorable in HIV-negative populations since some studies suggested that different treatments lead to lower recurrence rate in HIV negative, but definite data are lacking [36, 37].

## Conclusions

The administration of imiquimod can be considered as a safe off-label treatment for intra-anal and perianal HSILs, particularly for patients looking to avoid more invasive procedures. Its ability to induce lesion regression, modulate immune responses, and potentially clear underlying HPV infections makes it an attractive alternative or adjunct to surgical interventions. However, its efficacy is variable, especially in high-grade lesions and immunocompromised individuals, and recurrence rates remain high. Also, comparison between studies is difficult due to their heterogeneity and dishomogeneous data. Adverse events are frequent but mainly low-grade and should be monitored. Further research is needed to refine its use, particularly in combination with other therapies and in immunocompromised populations. Furthermore, in the pursuit of increasingly personalized therapy, new biomarkers to predict treatment response must be identified.

## Key References

- Gopalani SV, Senkomago V, Rim SH, Saraiya M. Human papillomavirus-associated anal squamous cell carcinoma: sociodemographic, geographic, and county-level economic trends in incidence rates-United States, 2001 - 2019. J Natl Cancer Inst [Internet]. 2024 Feb 8;116(2):275–82. Available from: http://www.ncbi.nlm.nih.gov/pubmed/37851397.
  - The study provides insights into how factors such as socioeconomic status and geographic location influence the occurrence of HPV-related anal carcinoma in the United States.
- Clifford GM, Georges D, Shiels MS, Engels EA, Albuquerque A, Poynten IM, et al. A meta-analysis of anal cancer incidence by risk group: Toward a unified anal cancer risk scale. Int J Cancer [Internet]. 2021;148(1):38–47. Available from: http://www.ncbi.nlm.nih.gov/pubmed/32621759
  - This study provides robust and comparable estimates of anal cancer incidence across various high-risk groups, and aim to inform prioritization and standardization in anal cancer prevention and research initiatives.
- Palefsky JM, Lee JY, Jay N, Goldstone SE, Darragh TM, Dunlevy HA, et al. Treatment of Anal High-Grade Squamous Intraepithelial Lesions to Prevent Anal Cancer. N Engl J Med [Internet]. 2022 Jun 16;386(24):2273–82. Available from: http://www.nejm.org/doi/10.1056/NEJMoa2201048
  - The study is the first RCT that found that treating anal HSIL significantly reduced the incidence of anal cancer among high-risk populations, such as individuals living with HIV. These findings support the implementation of treatment strategies for anal HSIL to prevent anal cancer in high-risk groups.
- Trutnovsky G, Reich O, Joura EA, Holter M, Ciresa-König A, Widschwendter A, et al. Topical imiquimod versus surgery for vulvar intraepithelial neoplasia: a multicentre, randomised, phase 3, non-inferiority trial. Lancet [Internet]. 2022 May;399(10337):1790–8. Available from: https://linkinghub.elsevier.com/retrieve/pii/S014067362200469X
  - This RCT found that imiquimod is a viable alternative to surgery, offering a non-invasive treatment option for HPV-related vulvar squamous intraepithelial lesions.

## Data Availability

No datasets were generated or analysed during the current study.
